# The Impact of Executive Functions on Metaphonological Skills: Correlation and Treatment Implication for ADHD Children

**DOI:** 10.3390/jcm15020906

**Published:** 2026-01-22

**Authors:** Adriana Piccolo, Margherita La Fauci, Carmela De Domenico, Marcella Di Cara, Alessia Fulgenzi, Noemi Mancuso, Lilla Bonanno, Maria Tresoldi, Rosalia Muratore, Caterina Impallomeni, Emanuela Tripodi, Francesca Cucinotta

**Affiliations:** 1 IRCCS Centro Neurolesi “Bonino Pulejo”, 98124 Messina, Italy; adriana.piccolo@irccsme.it (A.P.);; 2 Otorhinolaryngology and Auditory Microsurgery Unit, Department of Experimental Medical-Surgery, Specialist and Odontostomatologica Science, University of Messina, 98125 Messina, Italy

**Keywords:** ADHD, executive function, phonological awareness, metaphonological skills, language development, personalized intervention

## Abstract

Attention-deficit/hyperactivity disorder (ADHD) is a neurodevelopmental disorder frequently associated with impairments in executive functions (EF). These deficits have been linked to difficulties across various cognitive domains, including metaphonological skills (MS), essential for phonological awareness and processing abilities. **Background/Objectives**: This pilot study examines the correlations between EF and MS in ADHD children. **Methods**: A total of 84 children aged 6–14 years, diagnosed with ADHD and an IQ ≥ 70, were assessed using the NEPSY-II test to evaluate executive functions and the Assessment of Metaphonological Skills Test to assess phonological processing abilities. **Results**: Correlational analyses and multiple regression models were employed to explore the relationships between EF and MS, focusing on attention, cognitive flexibility, and response inhibition. Rhyme was positively correlated with processing speed and negatively correlated with response inhibition. Phonemic segmentation was significantly related to auditory attention and response inhibition. Age emerged as a significant predictor of phonemic synthesis and final syllable deletion, consistent with the developmental maturation of executive and phonological abilities. **Conclusions**: The findings suggest that deficits in executive functioning in ADHD children are closely linked to metaphonological abilities, which play a crucial role in the acquisition of reading and writing skills. Integrating EF training into phonological interventions can help reduce learning difficulties and improve cognitive and language outcomes.

## 1. Introduction

Attention-deficit/hyperactivity disorder (ADHD) is a neurodevelopmental condition characterized by inattention, hyperactivity, and/or impulsivity that interferes with personal functioning [1], with a prevalence estimate of about 3.4–9.4% in children aged 3–12 years [2,3]. ADHD is often associated with a substantial burden for individuals and their families, and may negatively affect academic performance, social interactions, and perceived quality of life [4,5]. This common disorder exhibits significant clinical heterogeneity, reflecting individual differences in genetic, environmental, and neurophysiological processes [6]. Moreover, several neuropsychiatric conditions often co-occurring during life-course [7,8] including Language Disorders (LD) in 40–50% of cases [9,10,11,12], and Specific Learning Disorder (SLD) in 25–40% [8,13].

Neuropsychological studies have highlighted different cognitive deficits in ADHD, particularly in executive functions (EF) [14,15,16,17,18,19]. Specifically, in ADHD, common EF deficits include working memory, planning, and response inhibition [20,21,22], often associated with worse academic and literacy outcomes [23,24].

EF encompasses high-order processes essential for daily activities [25], also influencing language abilities [26], early literacy and numeracy [27,28,29] and academic abilities [30,31]. The literature confirms an important relationship between EF and language development [32]. Core EF components, such as working memory, inhibitory control, and cognitive flexibility, support essential processes involved in language acquisition and comprehension, by helping children manage attention, retain verbal information, and adapt their communicative responses to context [25,32]. For example, verbal working memory facilitates the temporary storage and manipulation of phonological information, essential for understanding and producing language [33,34]. Inhibitory control helps children focus on relevant linguistic input and suppress irrelevant interpretations [35], while cognitive flexibility enables them to adapt language use to different communicative contexts [36]. Furthermore, growing evidence indicates that executive function (EF) abilities are involved in supporting specific linguistic processes, such as phonological awareness (PA) and metaphonological skills (MS) [37,38], emphasizing the interplay between executive control mechanisms and language-related domains [39,40]. However, although they are closely related, PA and MS represent distinct constructs. PA refers specifically to the ability to recognize and manipulate the sound structure of language (e.g., rhymes, syllables, phonemes) and is widely recognized as a proximal predictor of literacy acquisition [41,42]. MS, in contrast, represents a broader set of metalinguistic abilities that include PA but also extends to more complex operations, such as phonemic transformations, syllabic manipulation, and reflection on higher-level linguistic structures [43]. In this framework, PA can be regarded as a specific component within the broader domain of MS.

Both these cognitive processes mature during the preschool years and tend to become increasingly refined during early childhood [44]. PA tasks often require attention and working memory, highlighting the close interdependence between EF and phonological abilities [45,46]. Similarly, MS may also be compromised in the presence of EF deficits, suggesting a direct link between these cognitive domains [45,47,48]. Both phonological awareness and metaphonological skills are funda-mental for the development of expressive language [49] and represent key predictors of reading, writing, and overall literacy development [38,50,51].

However, the relationship between EF and MS remains insufficiently explored in populations commonly characterized by EF deficits, such as ADHD children.

Although comorbidity between ADHD and SLD is frequently observed and contributes to the complexity of clinical profiles [52,53], the present study focused specifically on children with ADHD children without a diagnosis of SLD. According to the DSM-5, Specific Learning Disorder is characterized by persistent difficulties in reading, writing, and/or mathematics that significantly interfere with academic achievement and are not better explained by intellectual disability, sensory deficits, or inadequate educational instruction [1]. This choice was intended to isolate the contribution of EF to MS, without the confounding influence of learning disorders, in which phonological processing difficulties constitute a core trait that cannot be primarily traced back to executive deficits.

To date, research investigating the association between MS and EF in ADHD is still limited. On the one hand, some studies suggest that ADHD children show poorer MS performance compared to typically developing peers [54,55,56,57]. On the other hand, other studies did not identify distinct MS deficits in ADHD when compared with children with LD, raising questions about whether these impairments are specific to ADHD [58,59].

Moreover, it remains relevant to further investigate this topic, as a persistent deficit in MS during childhood and adolescence may result in disadvantages in academic achievement, thus highlighting the need for identification as well as targeted and timely interventions [60].

This pilot study investigates the relationship between EF domains and MS in ADHD children. Specifically, we examined whether distinct EF domains were statistically correlated with MS components, in order to identify which executive processes are more closely linked to phonological processing in this population. Moreover, we aimed to examine whether age and IQ, included as covariates, influenced the relationship between EF and MS. To our knowledge, this is the first study that explores this correlation in this specific population.

## 2. Materials and Methods

### 2.1. Study Design

The pilot study was reviewed and approved by the Ethics Committee of the IRCCS Bonino Pulejo of Messina (approval number 15/2019); written informed consent was obtained from both parents and/or a legal representative of the patient. Every procedure involving human subjects was carried out in accordance with the Helsinki Declaration and the ethical standards of the institutional and/or national research committee. After verifying the inclusion criteria, participants undergo a comprehensive set of standardized measures grouped into three main cognitive domains: IQ, EF, and MS. This organization reflects the underlying theoretical framework of the study, which assumes these domains to be distinct yet interacting components of the targeted skill set, to examine the relationship between EF and MS, and to control for the potential confounding effects of age and IQ in this association. Given that both IQ and age significantly influence the development of executive functions [61], they were included as covariates in this pilot study. Age was controlled to account for the developmental progression of executive and phonological skills throughout the school years [62], while IQ was included to control the effects of general cognitive abilities on task performance. In the analysis, variables from each domain will be entered into separate models to test. Participants were consecutively enrolled at the Istituto di Ricovero e Cura a Carattere Scientifico (IRCCS) Neurolesi “Bonino Pulejo” in Messina, Italy, between February 2021 and March 2024. Children were recruited among those referred to the Child Neuropsychiatry Unit for clinical evaluation. All families of eligible children were informed about the study and invited to participate during their routine diagnostic assessment.

### 2.2. Inclusion Criteria

Inclusion criteria were (a) a primary diagnosis of ADHD according to the DSM-5 [1], not attributable to another medical or neurological condition; (b) age range between 6 and 14 years; (c) Intellectual Quotient (IQ) ≥ 70; (d) EF impairment, assuming the presence of a below-average (≤7 scalar score or ≤11 percentile) result in at least one task of Attention and Executive Functioning subtests of the NEPSY-II test as threshold. Participants with co-occurrent disorders, especially language impairment and LD, or other medical disorders such as epilepsy, visual and auditory sensory deficits, traumatic brain injury, or genetic syndromes, were excluded based on documented clinical diagnoses, reported in the child’s medical and confirmed by the neuropsychiatric team during enrollment, according to DSM-5 diagnostic criteria.

### 2.3. Measures

Standardized and validated instruments for the Italian population were used for data collection. The cognitive level was assessed using the Wechsler Intelligence Scales for Children (WISC-IV) [63]; EF was evaluated with NEPSY-II [64] and Tower of London [65]. Assessment of Metaphonological Skills Test (AMS) [66] was used to metaphonological skills Professionals qualified to administer these scales administered all the tests. Each child was evaluated in a quiet clinical setting across two sessions of approximately 60 min each. Breaks were provided as needed to minimize fatigue. All tests were administered following standardized procedures according to their respective manuals.

### 2.4. Executive Function

Executive functions were investigated with two standardized psychological tests: Nepsy II and Tower of London.

The NEPSY^®^ 2th Edition is a comprehensive neuropsychological test consisting of 32 subtests for assessment with preschoolers, children, and adolescents. In particular, we choose n. 3 subtests to measure attentional control, working memory, cognitive flexibility, fluency, performance monitoring, and response inhibition.

- Auditory Attention (AA, 5–16 years old) assesses precisely selective auditory attention and the child’s ability to sustain attention over time. The child listens to a digital audio file in which a recorded voice lists random words and words related to actions to be performed. The child is presented with a visual array of printed pictures and is instructed to touch the corresponding image (i.e., picture) each time the target word is heard. Each correct response was counted for the AA total score if performed within the two-second interval. Specifically, this task evaluates mainly auditory attention and sustained attention.
- Response Set (RS, 5–16 years old) assesses the child’s skills in modifying a learned response set by adopting a new and more complex one that includes congruent and incongruent responses. Before starting to listen to the digital audio file, the evaluator asks to touch a figure arbitrarily assigned to a specific word (e.g., “When you hear RED, touch the YELLOW circle”). Each correct response was counted for the RS total score if performed within the two-second interval. Specifically, this task evaluates mainly: cognitive flexibility, auditory attention, sustained attention, working memory, performance monitoring, and response inhibition/impulsivity.
- Inhibition (ages 5–16 years old) assesses the child’s ability to inhibit automatic responses in favor of novel ones and consists of three subtests: condition A (Naming), condition B (Inhibition), and condition C (Switching). In the first condition (Naming), shapes or arrows are shown to the child and he must name the shape or arrow’s direction quickly. Naming was used to evaluate fluency, performance monitoring, processing speed, and response inhibition.

In the second condition (Inhibition), the child inhibits the automatic naming response and instead provides the opposite name (i.e., named circle when a square is seen) as soon as possible, assessing the cognitive flexibility, performance monitoring, and response inhibition.

Finally, in the third condition, the child alternates between naming the correct response or the opposite response depending on the color of the stimulus (i.e., if the shape or arrow were white the child had to name the opposite) as soon as possible. Switching condition measures cognitive flexibility, working memory, performance monitoring, and response inhibition. All the conditions measured accuracy and speed of performance.

The Tower of London Test measures the ability of strategic reasoning, mental planning, and monitoring skills involved through problem-solving tasks [67]. Each item presents three balls of different colors (red, yellow, blue), positioned on three sticks of varying heights. The objective is to achieve the correct configuration according to a provided model, within a predetermined number of moves indicated by the experimenter. The test’s structure consists of 12 items, organized by complexity: the first two items can be completed in two moves, the next two in three moves, four items in four moves, and the final four items in five moves.

Performance was evaluated through different scores: the total score of correct answers, the number of moves taken for each attempt, the count of rule violations, the decision time, the execution time, and the total time. The total score is calculated based on correct answers across all problems. This score reflects the subject’s global planning ability and their capacity to achieve a goal instead of possible performance errors. Furthermore, correct execution involves the use of working memory and the ability to adapt to different schemes, which necessitate inhibiting perseverative responses.

The number of moves indicates how many moves the subject employs throughout the entire test; it serves as valuable data for analyzing the difficulty in planning, problem-solving, or response inhibition, and underlies the possible presence of perseverations. The recording of rule violations provides insights into the subject’s ability to understand and retain the presented rules, inhibit impulsive responses, performance monitoring, and attentional control.

Decision time represents an important metric for understanding impulsive behavior; it is calculated as the sum of the decision times for all trials, recorded until the moment of physical execution of the task. Execution time is calculated as the time from the beginning to the end of a task’s physical execution and is an indirect measure of impulsivity, performance monitoring, working memory, and motor coordination. The total time is obtained by summing the previous two times and reflects the overall speed with which the subject completed the test. All executive function subtests included in the study, together with the corresponding cognitive domains assessed, are reported in Table 1.

### 2.5. Assessment of Phonological Processing Skills

Phonological processing skills have been evaluated with the AMS. This Italian standardized test assesses the development of MS, particularly the ability to discriminate, fuse, elide, and manipulate phonological or syllabic material. Specifically, the following tests were administered:

- Rhyme reconnaissance: This test requires the recognition of rhymes, understood as a simple classificatory skill in which the child’s lexical ability is most involved. It is proposed to use visual support to the words presented orally by the examiner, i.e., four pictures including one stimulus word, one target, and two distractors.
- Verbal fluency with phonemic facilitation: this test is required to elicit the greatest number of words beginning with a given phoneme in one minute. This task provides information about both classification processes based on phonemic analysis and retrieval ability from the lexical storehouse.
- Phonemic synthesis: The task involves the identification of a word that results from the fusion of a sequence of phonemes presented orally. For example, after hearing the sounds /c/—/a/—/t/, the child is expected to blend them together and produce the word “cat”.
- Phonemic segmentation: The examiner pronounces a word aloud, and the child is asked to orally segment the word into its individual phonemes The child’s response is verbal, and no written stimulus is provided.
- Deletion of initial syllable: this test evaluated the child’s ability to perform operations on the phonological structure of language, thus on the ability to “manipulate” phonological material. The child must delete the initial segment of the word.
- Deletion of the final syllable the child must delete the final segment of the word and be able to reproduce the target word without the final syllable. Both the examiner’s question and the child’s answer are verbal, and no written stimulus is provided.
- Spoonerism: in this task, the child had to invert the initial phoneme of two words, providing two new ones. This test is particularly difficult, requiring considerable ability in phonemic synthesis and analysis, and can only be administered starting from the second grade of primary school. For example, after hearing the pair “cat—dog”, the correct response would be “dat—cog”

Each subtest consists of 15 items, except for the phonemic verbal fluency task, which includes three open-response items. Items are scored as 1 for correct and 0 for incorrect responses, except for the spoonerism task, which requires complete phonemic transformation to receive credit, and the phonemic verbal fluency task, which is scored based on the total number of correct words produced within the time limit.

All these items collectively describe the three macro-areas of phonological processing, summarized in Table 2.

### 2.6. Statistical Analysis

A nonparametric analysis was carried out because the results of the Shapiro normality test indicated that most of the target variables were not normally distributed. Numerical data are presented as medians and interquartile ranges, given that most variables did not meet normality assumptions. Correlations among variables were assessed using Spearman’s coefficient, specifically examining the relationships between metaphonological variables and executive-function measures. All NEPSY-II indicators were analyzed as age-corrected scaled scores, for which higher values reflect better performance (including the Naming, Inhibition, and Switching conditions); therefore, positive correlations indicate that better EF performance is associated with higher metaphonological scores. Tower of London indices were analyzed as T-scores, and NEPSY-II error/time indices were expressed as standardized scores; in all cases, higher scores reflect better performance (e.g., fewer errors/violations and/or faster performance depending on the index). Given the exploratory purpose of the correlation analysis and the relatively large number of comparisons, we reported uncorrected *p*-values (threshold *p* < 0.05) without applying formal correction for multiple comparisons, in order to reduce the risk of type II errors due to over-conservative adjustment. Correlations were interpreted with caution and used to guide variable selection for subsequent regression modeling. Because multiple bivariate tests may inflate type I error, these associations were treated as hypothesis-generating and were not interpreted as confirmatory evidence. To mitigate the risk of selecting spurious predictors from bivariate screening, we complemented the exploratory AIC-based models with hierarchical (block-wise) regressions testing the incremental contribution of EF domains beyond age and IQ (Supplementary Table S2). Age and IQ were included as covariates to account for developmental and general cognitive influences on both EF and MS performance, and to evaluate whether EF subcomponents explain MS outcomes beyond these factors. Based on these results, methodological variables showing the strongest and most consistent associations with executive function (namely, phonemic synthesis and deletion of the final syllable) were selected as outcome variables for sub-sequent regression analyses. For each of these outcomes, we specified an initial full multiple linear regression model that included age, IQ, and all executive function variables that were significantly or marginally associated with the outcome in the bivariate analyses (*p* < 0.10). Multiple linear regression analyses were then conducted using a backward stepwise elimination procedure, with variable selection based on the Akaike Information Criterion (AIC) in order to identify the most parsimonious final models. To better estimate the independent contribution of EF subcomponents while controlling for shared variance across EF measures, we additionally performed hierarchical (block-wise) multiple regression analyses. Age and IQ were entered in the first block, followed by EF indicators entered in successive blocks reflecting EF subcomponents (processing speed/monitoring: NEPSY Naming time; attention: Auditory Attention; cognitive flexibility/monitoring: Response Set; inhibitory control: Inhibition time; switching: Switching time; planning/problem solving: Tower of London rule violations and total score). Models were estimated on complete cases to ensure identical sample size across blocks (*n* = 44). For hierarchical models, we report R^2^, ΔR^2^, and nested-model F-tests for each block; multicollinearity was checked using variance inflation factors (VIF), and final-model coefficients were computed with HC3 robust standard errors. All analyses were conducted using the open-source software package R (version 4.2.2; R Foundation for Statistical Computing, Vienna, Austria). A 95% confidence level was established, with an alpha error of 5%. Statistical significance was set at *p* < 0.05.

## 3. Results

**Figure 1:**
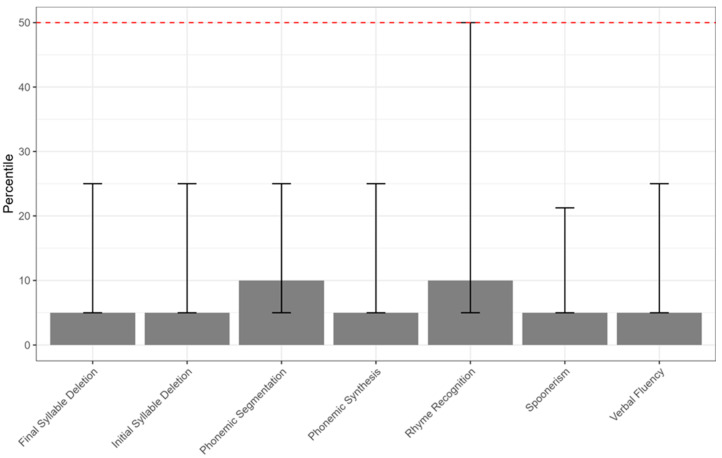
Median percentile scores on the Assessment of Metaphonological Skills (AMS) subtests. Error bars represent the interquartile range (Q1–Q3). The dashed horizontal line indicates the normative reference value (percentile = 50).

**Figure 2:**
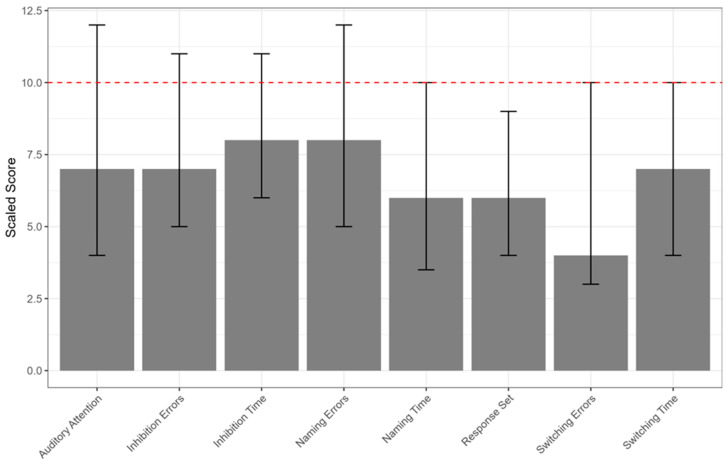
Median scaled scores on NEPSY-II subtests. Error bars represent the interquartile range (Q1–Q3). The dashed horizontal line indicates the normative average (scaled score = 10).

**Figure 3:**
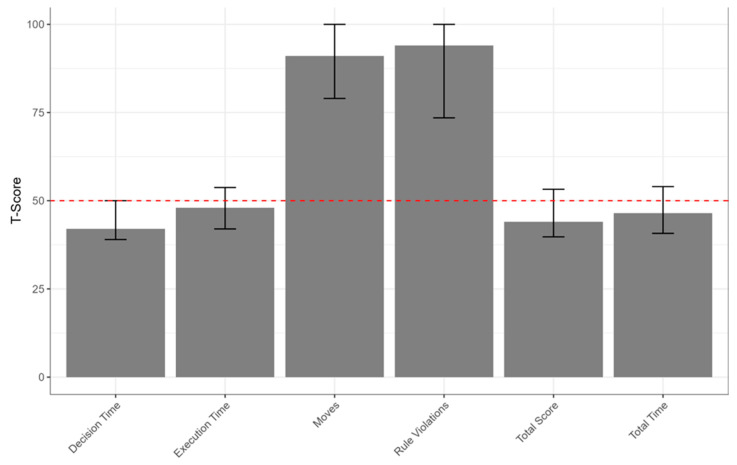
Median T-scores of Tower of London subtests. Error bars represent the interquartile range (Q1–Q3). A dashed horizontal line indicates the normative reference value (T = 50).

A total of 92 children were initially enrolled. After applying the exclusion criteria, 8 participants were excluded, resulting in a final sample of 84 children (mean age = 7.94 ± 1.67 years; 36 males, 42.9%, and 48 females, 57.1%). Demographic characteristics were reported in Table 1. Clinical characteristics of the sample were reported in Table 3 and Figure 1, Figure 2 and Figure 3. In particular, we showed the median of the sub-score of the assessment of AMS (Figure 1), NEPSY II (Figure 2), and TOL (Figure 3).

The Spearman correlation analysis revealed significant positive correlations between Rhyme and NEPSY-II Naming scaled score (r = 0.23; *p* = 0.04), as well as with NEPSY-II Switching Errors scaled score (r = 0.3; *p* = 0.003). Additionally, a significant negative correlation was found between Rhyme and Tower of London Rule Violations T-score (r = −0.33; *p* = 0.005). Verbal fluency with phonetic facilitation was positively correlated with NEPSY-II Naming scaled score (r = 0.25; *p* = 0.02) and showed a trend with the response set (r = 0.25; *p* = 0.05). Significant positive correlations were also observed between phonemic segmentation and auditory attention (r = 0.23; *p* = 0.04), response set (r = 0.28; *p* = 0.03), and NEPSY-II Naming scaled score (r = 0.27; *p* = 0.01), while a negative correlation was found with rule violation (r = −0.27; *p* = 0.02). Deletion of the final syllable was positively correlated with the response set (r = 0.31; *p* = 0.01) and negatively correlated with rule violation (r = −0.28; *p* = 0.02). Trends were noted between Deletion of the final syllable and age of the participants (r = 0.2; *p* = 0.07), auditory attention (r = 0.22; *p* = 0.05), and NEPSY-II Naming scaled score (r = 0.21; *p* = 0.06). Finally, trends were observed between phonemic synthesis and age of participants (r = 0.2; *p* = 0.07), NEPSY-II Naming scaled score (r = 0.2; *p* = 0.07), deletion of initial syllable and response set (r = 0.25; *p* = 0.05), and switch errors (r = 0.26; *p* = 0.06) (Table S1).

### Multiple Regression Analysis

Multiple regression analyses (exploratory AIC-based models) indicated that, after accounting for covariates, age was retained in the final reduced models, whereas EF predictors were not consistently retained (Table 3 and Table 4). Given that MS outcomes were scored as correct-performance indices (higher scores indicate better performance), higher age was associated with higher metaphonological scores. These models were intended to evaluate whether EF measures explain MS outcomes beyond age and IQ, not to predict age. In the initial (full) regression models, age, IQ, and EF measures were entered as predictors; EF measures showed only non-significant or trend-level effects, and IQ did not contribute significantly. Accordingly, the stepwise AIC-based procedure retained age in the final reduced models (Table 4 and Table 5).

In addition, to account for the overlap among EF measures and estimate the independent contribution of EF subcomponents, we performed hierarchical (block-wise) regression models (Supplementary Table S2). EF blocks did not provide significant incremental variance beyond age and IQ for either outcome (all nested-model tests *p* > 0.05). In the fully adjusted hierarchical model, age remained significantly associated with phonemic synthesis (*p* = 0.010; HC3 robust SEs), whereas no significant predictors were observed for final syllable deletion. This suggests that the age effect was robust for phonemic synthesis, while the association with final syllable deletion was less stable once mutual adjustment across EF domains was applied. Therefore, while several EF measures showed bivariate associations with MS, these relationships did not remain independently significant after mutual adjustment across EF domains and covariates.

Overall, the regression findings should be interpreted with caution: effect sizes were modest, several coefficients only reached trend-level significance, and the complete-case sample available for multivariable modeling was relatively small (*n* = 44). As such, these models are best viewed as preliminary and hypothesis-generating rather than providing robust confirmatory evidence.

## 4. Discussion

### 4.1. Executive Function-Metaphonological Skill Associations in ADHD

This pilot study investigated the associations between EF and MS in ADHD children, with a particular focus on which EF components are most closely linked to different aspects of phonological processing. The wide age range of the sample (6–14 years) enabled us to examine these associations across different stages of development.

Our analyses revealed significant correlations between EF performance and MS, consistent with and extending previous findings [38]. Among the MS components, phoneme segmentation and rhyme recognition emerged as the most strongly associated with EF measures. The positive associations between phoneme segmentation, auditory attention, and the response set task suggest that children with more efficient attentional control tend to show better phonological processing skills. These tasks rely on listening and word recognition abilities, both of which appear to require sustained auditory attention, a key prerequisite for the development of MS [68,69].

Performance on the response set task appears to rely on inhibitory control and response monitoring, EF components that support the development of early linguistic abilities [70]. Moreover, difficulties in self-monitoring are known to affect speech production processes, as shown in conflict-based models of speech monitoring [71]. Although the association did not reach statistical significance, a trend was observed indicating that better response set performance was linked to higher verbal fluency, suggesting a possible relationship between self-monitoring and fluent language production. This interpretation aligns with prior evidence highlighting the involvement of EF in supporting language fluency [70].

The positive correlations between auditory attention and phoneme segmentation further suggest that children who sustain attention during phonological tasks tend to perform more accurately on seg-mentation tasks [72]. This is a clinically relevant observation, as segmentation skills are strongly associated with reading [73] and writing development [74]. Moreover, phoneme segmentation was negatively associated with rule violations, which reflect challenges in inhibitory control and planning. A similar pattern was observed for rhyme recognition, supporting the hypothesis that impulse control plays an important role in phonological processing [75]. Comparable inhibitory control pro-files have been documented in ADHD children with reading difficulties [76].

Rhyme recognition was also positively associated with NEPSY-II Switching Errors scaled score, reinforcing its relationship with cognitive flexibility and self-monitoring [38,70], two EF components frequently impaired in ADHD [19]. This result is particularly notable because rhyme recognition is considered a global metaphonological skill, typically acquired independently of formal reading instruction [77].

Finally, the NEPSY-II Naming scaled score was significantly associated with both phoneme segmentation and rhyme recognition. This finding suggests that more efficient rapid naming/visual–verbal processing, as captured by the NEPSY-II Naming condition, is linked to better metaphonological performance. Processing speed deficits are well documented in dyslexia [78] and are also common in ADHD, where RAN performance is associated with reading fluency even after controlling IQ, basic reading skills, ADHD symptom severity, and age [79]. The observed association between verbal fluency and NEPSY-II Naming scaled score suggests a shared underlying mechanism linking EF to language performance.

Taken together, these results indicate that inhibitory control, performance monitoring, and sustained attention are the EF components most closely associated with MS, particularly phoneme segmentation and rhyme recognition.

### 4.2. Developmental Variance and Overlapping Task Demands

EF–MS associations were evident at the bivariate level but attenuated after mutual adjustment, suggesting shared developmental variance and overlapping task demands. However, in multivariable models that jointly controlled for age, IQ, and overlapping EF domains, EF indicators did not provide robust independent contributions beyond age, suggesting that the observed EF–MS correlations may primarily reflect shared developmental variance and task demands. Importantly, the attenuated and unstable EF effects observed in the multivariable models, together with wide confidence intervals around several coefficients, indicate that the present study was not sufficiently powered to draw firm conclusions regarding the unique predictive value of specific EF components. In this sense, the regression analyses should be regarded as exploratory and interpreted with particular caution.

From a clinical perspective, executive function impairments represent a clinically relevant characteristic of ADHD and are commonly observed across different cognitive and behavioral domains, particularly those related to attentional regulation and impulse control [15,18]. Within this broader cognitive profile, metaphonological skills appear to be closely intertwined with executive functioning, as they rely on inhibitory control, sustained attention, and performance monitoring processes [32,45]. The associations observed between executive functions and metaphonological skills in the present study further support the clinical relevance of MS as part of the cognitive profile associated with ADHD.

### 4.3. Role of Age and IQ in Metaphonological Performance

A secondary objective of this pilot study was to investigate whether age and IQ covary with the EF–MS associations. Age was included as a covariate to account for the well-documented developmental trajectory of metaphonological skills across school years [80], whereas IQ was considered to control for general cognitive abilities that can influence phonological performance [81]. In regression analyses, age—rather than IQ—accounted for a meaningful portion of variance in the selected MS outcomes, particularly phonemic synthesis. Given that MS outcomes were scored as correct-performance indices (higher scores indicate better performance), higher age was generally associated with higher metaphonological scores, consistent with developmental maturation. Notably, age effects appeared to be task-dependent: while phonemic synthesis showed the most robust association with age in multivariable models, the association with final syllable deletion was less stable once covariates and overlapping EF domains were jointly controlled (Supplementary Table S2). IQ did not contribute significantly to the models. This pattern suggests that developmental factors may influence some more analytically demanding MS components, whereas the EF–MS associations observed in bivariate analyses may partly reflect shared developmental variance and task demands rather than strong independent EF effects in this pilot sample. Although phonological skills typically improve with age [82], specific components such as rhyme recognition and phoneme segmentation—those most strongly related to EF in the bivariate analyses—may still rely heavily on inhibitory control and attentional processes. Overall, while age-related maturation likely plays a role in shaping certain aspects of MS, the present study was not powered to test non-linear developmental trends or moderation effects (e.g., age × EF). Future studies with larger samples should explicitly examine whether EF–MS associations differ across narrower developmental bands and whether age moderates the contribution of specific EF components to MS outcomes.

### 4.4. Neurocognitive Interpretation and Clinical Implications

From a neurocognitive perspective, while phonological awareness is fundamentally a linguistic ability, some MS tasks seem to require a higher degree of regulatory and control processes—core components of EF. Previous research has shown that phonological processes, more than semantic ones, are closely linked to EF [70]. In particular, difficulties in detecting phonological errors have been associated with damage to the dorsolateral prefrontal cortex, a brain region essential for executive control [83,84].

Taken together, these observations indicate that successful performance on MS tasks depends not only on explicit linguistic knowledge but also on domain-general cognitive resources—such as working memory, sustained attention, and executive control—which support the retrieval, manipulation, and monitoring of phonological representations [85]. Neuroanatomical evidence further supports this view, showing that EF impairments are associated with reduced reading fluency and altered activation patterns within the left-hemisphere reading network [27].

Overall, these results highlight the clinical importance of assessing MS early in children with EF impairments. Incorporating phonological awareness assessment into ADHD evaluations could help identify early risk markers for SLD. Indeed, rhyme recognition has been identified as one of the most reliable predictors of reading and writing disorders [86], and phonemic segmentation has been shown to predict reading skills [87].

The present findings underscore the importance of including MS assessment in clinical practice with ADHD populations. Elucidating the EF–MS relationship could provide a critical framework for de-signing targeted interventions to optimize academic performance. Early interventions addressing EF components (e.g., impulse control, attention, performance monitoring) alongside phonological skills may enhance literacy acquisition. Although interventions focused on academic prerequisites can im-prove outcomes, some skills, such as rhyme recognition, may remain relatively resistant to change [88]. Cognitive training combined with targeted phonological instruction could therefore represent a promising strategy to support learning outcomes in ADHD children.

### 4.5. Limitations

Although this pilot study provides valuable insights, several limitations should be acknowledged. First, analytical skills, which are significantly influenced by educational level, may have been affected by the heterogeneity of the sample, as it was not stratified by educational background. Another important limitation concerns the broad age range of the sample. Both executive functions and meta-phonological skills follow distinct developmental trajectories, and it is plausible that differences be-tween younger and older children may modulate the strength of EF–MS associations [89,90]. Although age was statistically controlled as a continuous covariate in our analyses, future studies should stratify participants into narrower developmental subgroups to better clarify whether, and how, these associations differ across developmental stages. A further limitation concerns the lack of systematic information on ADHD subtypes and severity profiles. Although all participants met DSM-5 diagnostic criteria for ADHD, subtype classification was not consistently available and was therefore not included in the analyses. Given that different ADHD subtypes can be associated with distinct executive and language profiles, future studies should explicitly examine whether FE-MS relationships differ across different ADHD presentations. Furthermore, information regarding pharmacological treatment was not systematically available and was therefore not considered in the present analyses. Finally, given the exploratory and pilot nature of the study, as well as the large number of comparisons conducted without correction for multiple testing, the present findings should be interpreted with caution. In addition, because the exploratory correlation matrix involved multiple comparisons and was used to inform regression modeling, there is an increased risk of false-positive findings and potential overfitting; therefore, the observed EF–MS associations should be considered hypothesis-generating and require confirmation in larger, independent samples using corrected and/or preregistered analytic strategies. Finally, the multivariable regression analyses were conducted on a reduced complete-case subsample and yielded relatively weak and unstable associations, with small effect sizes and several predictors only reaching trend-level significance. Consequently, these regression findings cannot be considered robust or confirmatory and should be interpreted with caution, primarily as a starting point for hypothesis generation to be tested in larger, adequately powered samples. Despite these limitations, the present study highlights significant correlations between executive functions and phonological skills in ADHD children, emphasizing the importance of focusing on these areas in assessments where language and/or executive function deficits are evident. As supported by numerous authors [91,92,93], the involvement of phonological skills in learning processes can depend on both phonological and non-phonological mechanisms. Given the widely recognized importance of phonological skills in the pre-requisites for school learning [94], it would be appropriate to consider them in the diagnostic process in subjects presenting executive function impairments.

Building these considerations, future research should incorporate additional cognitive measures, such as verbal memory span and rapid automatized naming, which may further influence participants’ performance and outcomes.

Many future directions can still be explored with a larger sample, such as testing the influence of gender and already acquired verbal skills. In addition, it would be worthwhile to test whether and how behavioral problems reported by parents or teachers might also influence a role on phonological skills. Future research could benefit from comparing ADHD subgroups with and without comorbid SLD, to clarify the specific role of comorbidity in shaping developmental trajectories. Additionally, longitudinal research would help clarify the developmental trajectories of these skills in ADHD children and their impact on academic achievement.

## 5. Conclusions

This pilot study provides preliminary evidence of a close relationship between executive functions and metaphonological abilities in ADHD children. The significant correlations observed suggest that executive function difficulties, which are commonly reported in ADHD, may be associated with weaker metaphonological skills. However, given the cross-sectional design and the relatively limited sample size, and the modest strength of the multivariable effects, these findings should be interpreted with caution. Within these limits, the results highlight the potential value of early assessment of executive functions and metaphonological-related abilities, which may support the identification of children at risk and inform targeted intervention strategies. Difficulties in these domains may represent vulnerability factors for later academic challenges, including an increased risk for learning disorders related to phonological processing. Longitudinal studies with larger, adequately powered samples are needed to clarify the developmental trajectories and causal relationships among executive functions, metaphonological skills, and academic outcomes in ADHD, and to confirm the relatively weak and unstable regression patterns observed in this pilot sample, which should be regarded as preliminary and hypothesis-generating.

## Figures and Tables

**Table 1:** Executive function measures included in the study, reporting the corresponding subtests and the main cognitive domains assessed.

Test	Subtest	Cognitive Domain
NEPSY-II	Auditory Attention	Selective and sustained attention
NEPSY-II	Response Set	Cognitive flexibility, response inhibition, working memory
NEPSY-II	Inhibition—Naming	Processing speed, fluency, response monitoring
NEPSY-II	Inhibition—Inhibition	Response inhibition, cognitive control
NEPSY-II	Inhibition—Switching	Cognitive flexibility, working memory, inhibition
Tower of London	Total score/time/errors	Planning, problem solving, monitoring, inhibition

**Table 2:** Item Evaluation of Metaphonological Skills Test (AMS), the three macro-areas of phonological processing and the corresponding metaphonological skills.

Item AMS	Metaphonological Awareness	Metaphonological Skills
Rhyme reconnaissance	global awareness	Sound Judgment task
Verbal fluency with phonemic facilitation	analytical awareness	
Phonemic synthesis	analytical awareness	Synthesis and Segmentation
Phonemic segmentation		
Deletion of initial syllable	analytical awareness analytical awareness	Manipulation
Deletion of final syllable		
Spoonerism	analytical awareness	

**Table 3:** Demographic description of the sample.

		*N*	Average (SD) or %
Sample		84	
Age			7.94 (1.667)
Gender	Male	48	57.1%
	Female	36	42.9%
Intellectual Quotient			89.8

**Table 4:** Results of multiple linear regression using age and executive function variables as predictors of metaphonological outcomes. Results from the final reduced model are shown (including trend-level predictors, where applicable).

Dependent Variables	Predictors	β	Std β	*p*-Value	Adjusted R^2^
Phonemic synthesis	Age	5.66	0.47	0.02	0.14
	Switching Time Scaled Score	1.99	0.40	0.09	
Deletion of the final syllable	Age	5.39	0.44	0.03	0.01
	Execution Time T-Score	−1.15	−0.64	0.07	

Legend: β = regression coefficient; Std β = standardized regression coefficient.

**Table 5:** Results of multiple linear regression using IQ and executive function variables as predictors of metaphonological outcomes. Results from the final reduced model are shown.

Dependent Variables	Predictors	β	Std β	*p*-Value	Adjusted R^2^
Phonemic synthesis	Age	5.84	0.48	0.04	0.17
Deletion of the final syllable	Age	7.28	0.59	0.02	0.05

Legend: β = regression coefficient; Std β = standardized regression coefficient.

## Data Availability

Data and materials related to this study are available upon reasonable request from the corresponding author.

## Supplementary Materials

The following supporting information can be downloaded at: https://www.mdpi.com/article/10.3390/jcm15020906/s1, Table S1. Spearman’s correlation coefficients (ρ) for all pairwise associations between metaphonological outcomes and executive-function measures. Table S2. Hierarchical (block-wise) multiple regression models examining the incremental contribution of executive-function (EF) subcomponents to metaphonological outcomes. Age and IQ were entered in Block 1, followed by EF indicators entered in successive blocks (processing speed/monitoring, attention, response set, inhibition, switching, and planning/problem solving). The table reports model R^2^, the incremental change in explained variance (ΔR^2^) at each step, and nested-model F-tests (*p*-values) for phonemic synthesis and final syllable deletion. Models were estimated on complete cases (n = 44); final-model coefficients were computed using HC3 robust standard errors.
