# Supplementary material for: The Impact of Executive Functions on Metaphonological Skills: Correlation and Treatment Implication for ADHD Children
Source: J Clin Med. 2026 Jan 22;15(2):906. doi: 10.3390/jcm15020906 (PMC12842362; doi:10.3390/jcm15020906)
Supplement: Supplementary file 1 [file jcm-15-00906-s001.zip › jcm-4036700-supplementary.pdf]

Table S1. Spearman's correlation coefficients (ϱ) for all pairwise associations between metaphonological outcomes and executive-function measures

Metaphonological Skill	Executive Function	Spearman rho	p-value
Rhyme Recognition	Auditory Attention	0,16	0,162
	Response Set	0,21	0,094
	NEPSY-II Naming scaled score	0,16	0,145
	Naming Errors	0,23	0,040
	Inhibition Time	-0,07	0,543
	Inhibition Errors	0,04	0,709
	NEPSY-II Switching Errors scaled score	0,30	0,030
	Switching Time	0,23	0,095
	Rule Violations	-0,33	0,005
	Decision Time	0,13	0,264
	Execution Time	0,02	0,867
	Total Time	0,03	0,811
	Total Score	0,14	0,239
	Moves	-0,17	0,168
Verbal Fluency	Auditory Attention	0,19	0,096
	Response Set	0,25	0,048
	NEPSY-II Naming scaled score	0,25	0,022
	Naming Errors	0,16	0,141
	Inhibition Time	0,13	0,244
	Inhibition Errors	0,10	0,364
	NEPSY-II Switching Errors scaled score	0,12	0,413
	Switching Time	0,14	0,295
	Rule Violations	-0,15	0,209
	Decision Time	0,02	0,870
	Execution Time	-0,04	0,716
	Total Time	-0,02	0,872
	Total Score	0,02	0,878
	Moves	-0,05	0,696
Phonemic Synthesis	Auditory Attention	0,11	0,317
	Response Set	0,17	0,192
	NEPSY-II Naming scaled score	0,20	0,075
	Naming Errors	0,11	0,318
	Inhibition Time	0,07	0,551
	Inhibition Errors	0,11	0,342
	NEPSY-II Switching Errors scaled score	0,20	0,153
	Switching Time	0,12	0,381
	Rule Violations	-0,21	0,081
	Decision Time	0,06	0,633
	Execution Time	0,04	0,731

	Total Time	0,08	0,509
	Total Score	0,01	0,911
	Moves	0,09	0,459
Phonemic Segmentation	Auditory Attention	0,23	0,036
	Response Set	0,28	0,028
	NEPSY-II Naming scaled score	0,27	0,015
	Naming Errors	0,17	0,131
	Inhibition Time	0,12	0,266
	Inhibition Errors	0,12	0,298
	NEPSY-II Switching Errors scaled score	0,16	0,256
	Switching Time	0,07	0,631
	Rule Violations	-0,27	0,025
	Decision Time	0,08	0,514
	Execution Time	-0,06	0,599
	Total Time	-0,03	0,788
	Total Score	0,08	0,483
	Moves	-0,12	0,312
Initial Syllable Deletion	Auditory Attention	0,13	0,243
	Response Set	0,25	0,053
	NEPSY-II Naming scaled score	0,13	0,235
	Naming Errors	0,03	0,770
	Inhibition Time	0,08	0,468
	Inhibition Errors	0,07	0,532
	NEPSY-II Switching Errors scaled score	0,26	0,060
	Switching Time	-0,01	0,946
	Rule Violations	-0,12	0,309
	Decision Time	-0,17	0,153
	Execution Time	0,06	0,592
	Total Time	0,06	0,628
	Total Score	-0,10	0,416
	Moves	0,09	0,451
Final Syllable Deletion	Auditory Attention	0,22	0,051
	Response Set	0,31	0,014
	NEPSY-II Naming scaled score	0,21	0,057
	Naming Errors	0,12	0,292
	Inhibition Time	0,12	0,287
	Inhibition Errors	0,18	0,108
	NEPSY-II Switching Errors scaled score	0,14	0,334
	Switching Time	0,06	0,677
	Rule Violations	-0,28	0,017
	Decision Time	0,00	0,991
	Execution Time	0,01	0,927

	Total Time	-0,03	0,820
	Total Score	0,03	0,801
	Moves	-0,04	0,738
Spoonerism	Auditory Attention	-0,01	0,964
	Response Set	0,11	0,671
	NEPSY-II Naming scaled score	0,13	0,597
	Naming Errors	0,22	0,385
	Inhibition Time	-0,08	0,758
	Inhibition Errors	0,05	0,840
	NEPSY-II Switching Errors scaled score	0,39	0,125
	Switching Time	0,06	0,829
	Rule Violations	-0,21	0,424
	Decision Time	0,06	0,808
	Execution Time	-0,01	0,969
	Total Time	0,12	0,632
	Total Score	-0,06	0,803
	Moves	-0,11	0,670

Table S2. Hierarchical (block-wise) multiple regression models examining the incremental contribution of executive-function (EF) subcomponents to metaphonological outcomes. Age and IQ were entered in Block 1, followed by EF indicators entered in successive blocks (processing speed/monitoring, attention, response set, inhibition, switching, and planning/problem solving). The table reports model R^2, the incremental change in explained variance (ΔR^2) at each step, and nested-model F-tests (p-values) for phonemic synthesis and final syllable deletion. Models were estimated on complete cases (n = 44); final-model coefficients were computed using HC3 robust standard errors.

(A) Outcome: Phonemic synthesis

Step (Block added)	R^2	ΔR^2	F (df1, df2)	p
Block 1: Covariates (Age + IQ)	0.31	—	—	—
Speed/Monitoring (NEPSY-II Naming scaled score)	0.35	0.03	2.23 (1, 40)	0.14
Attention (Auditory Attention)	0.36	0.01	0.70 (1, 39)	0.40
Response Set	0.36	0.00001	0.0006 (1, 38)	0.98
Inhibition (Inhibition time)	0.36	0.0004	0.02 (1, 37)	0.88
Switching (Switching time)	0.39	0.03	2.08 (1, 36)	0.16
Planning/Problem solving (TOL rule violations + total score)	0.42	0.03	0.87 (2, 34)	0.43

(B) Outcome: Fynal syllable deletion

Step (Block added)	R^2	ΔR^2	F (df1, df2)	p
Block 1: Covariates (Age + IQ)	0.22	—	—	—
Speed/Monitoring (NEPSY-II Naming scaled score)	0.22	0.0002	0.01 (1, 40)	0.92
Attention (Auditory Attention)	0.24	0.02	0.90 (1, 39)	0.35
Response Set	0.28	0.001	0.05 (1, 38)	0.83

Inhibition (Inhibition time)	0.24	0.005	0.25 (1, 37)	0.62
Switching (Switching time)	0.24	0.0003	0.02 (1, 36)	0.90
Planning/Problem solving (TOL rule violations + total score)	0.26	0.02	0.46 (2, 34)	0.63
